# Extrapulmonary Gastrointestinal Presentation of Coronavirus (COVID-19): A Case Report and Review of Literature

**DOI:** 10.7759/cureus.8104

**Published:** 2020-05-13

**Authors:** Faiza Chaudhry, Tejasvi Kainth, Nicole M Sakla, Gagandeep Singh, Valentin Marian

**Affiliations:** 1 Internal Medicine, Jersey City Medical Center, Jersey City, USA; 2 Language Access and Internal Medicine, Winnipeg Regional Health Authority, Winnipeg, CAN; 3 Radiology, Newark Beth Israel Medical Center and Children's Hospital of New Jersey, Newark, USA

**Keywords:** covid-19, diarrhea, coronavirus, angiotensin-converting enzyme 2 (ace-2)

## Abstract

Coronavirus disease 2019 (COVID-19) is currently the causative agent for a global health emergency and is predominantly associated with respiratory symptoms. In this case, a patient presented to the emergency department with gastrointestinal symptomatology without associated respiratory findings and was subsequently diagnosed with COVID-19 based on incidental findings from an abdominal computed tomography (CT) study. Given the patient’s lack of respiratory symptoms, diagnosis and treatment were ultimately delayed. During this global health crisis, an improved understanding of the various presentations of COVID-19 is paramount in an effort to initiate immediate treatment and prevent further transmission.

## Introduction

According to the World Health Organization (WHO), on January 21, 2020, a novel type of coronavirus called severe acute respiratory syndrome coronavirus 2 (SARS-CoV-2) was first isolated on January 7, 2020 [1]. The outbreak was associated with exposure within a seafood market in Wuhan, China. According to the WHO report, 282 total cases were reported in China (278 cases), Thailand (two cases), Japan (one case), and the Republic of Korea (one case) as of January 20th. Criteria used for defining severe illness were dyspnea, respiratory rate (RR) > 30 beats per minute (bpm), hypoxemia, and chest x-ray demonstration of multilobar infiltrates [1].

When comparing the WHO Situation Report-1 to Situation Report-73 on April 2, 2020, there were 896,450 confirmed cases of coronavirus disease-2019 (COVID-19), with 45,526 deaths reported worldwide due to the disease outbreak [2]. The total cases and the number of deaths caused by COVID-19 in the United States (US) on April 1st were 163,199 and 2,850, respectively [3]. Comparatively, the Situation Report-73 showed 187,302 confirmed cases and 3,846 deaths, respectively, on April 2, 2020 [2].

This data demonstrates that within 24 hours, 24,103 new cases and 996 deaths were reported in the US [2-3]. The staggering viral mortality is, therefore, increasing by the hour. In this case, we present a patient with atypical gastroenterological rather than respiratory symptoms for COVID-19 with the hopes that awareness and early management might be initiated in patients that would otherwise not be identified.

## Case presentation

**Figure 1:**
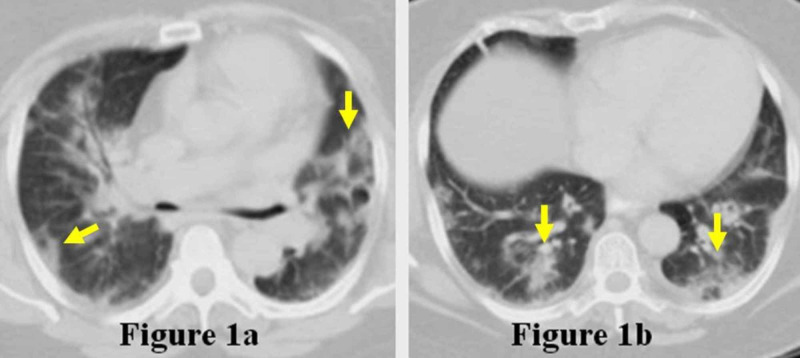
Initial Computed Tomography (CT) of the Abdomen Bibasilar peripheral ground-glass opacities (arrows) in superior (Figure 1a) and inferior (Figure 1b) slices from the initial CT abdomen examination.

We present the case of a 63-year-old obese Hispanic female with a past medical history only significant for rheumatoid arthritis and Sjogren's syndrome who presented to the emergency department with non-bloody diarrhea, nausea, vomiting, and right-sided abdominal pain radiating to her back for a week. The patient also reported having mild headaches but denied any cough or upper respiratory tract infection symptoms. While in the emergency department, the patient stated that she was dizzy. She was subsequently placed in observation for monitoring, where she was observed to have a persistent low-grade fever, measuring 100.9°Fahrenheit (F). Computed tomography (CT) of the abdomen was obtained and revealed patchy, peripheral multifocal ground-glass opacities at the lung bases (Figure 1a-b).

**Figure 2:**
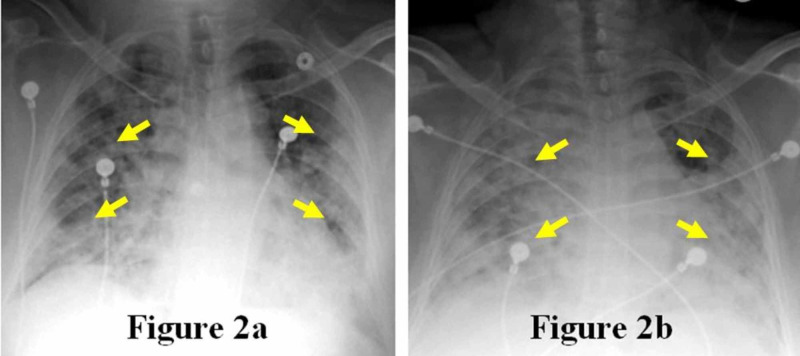
Initial and Subsequent Chest Radiographs Multifocal pneumonitis (arrows) on the initial radiograph (Figure 2a) and disease progression on the subsequent radiograph (Figure 2b).

On further investigation, the patient noted that she recently visited her sister in New York and that her sister also had chills, fever, and “pneumonia." The patient’s fever began two days after returning home from New York and she was discharged home after being evaluated at a nearby hospital at the time. Due to the ground-glass opacities seen on the abdominal CT examination, a fever for seven days, and a known history of a sick contact nine days prior, the patient was admitted for possible COVID-19 infection. Her low-grade fever of 100.9°F persisted for 24 hours in the hospital. The patient was initially started on lopinavir/ritonavir, ceftriaxone, and azithromycin. The patient ultimately tested positive for COVID-19 via nasopharyngeal swab. Subsequent chest x-rays showed progression of the ground-glass infiltrates (Figure 2a-b), and the patient eventually died after endotracheal intubation.

## Discussion

China reported cases of coronavirus within Wuhan, Hubei, China to the WHO on December 31, 2019 [4]. The causative organism was enveloped ribonucleic acid (RNA) betacoronavirus [5]. Currently, the disease is referred to as coronavirus disease 2019 (COVID-19) by the WHO [6]. The median age of presentation was 49 years, and the most common presenting complaints were fever (98%), cough (76%), and myalgia (44%) [7]. Other less common symptoms included headache (8%), diarrhea (3%), sputum production (28%), and hemoptysis (5%). Ground-glass opacities on CT scan of the chest were the most common radiologic finding in hospitalized patients.

Pan et al. observed that 50.5% of patients infected with COVID-19 reported gastrointestinal (GI) symptoms, including lack of appetite (78.6% of cases), diarrhea (34% of cases), vomiting (3.9% of cases), and abdominal pain (1.9% of cases), along with the presence of respiratory symptoms [8]. Three percent of the patients only reported GI symptoms without respiratory symptoms. GI symptoms became more pronounced as the severity of disease increased. Also, these patients had higher levels of liver enzymes, namely AST and ALT, longer prothrombin time, and lower monocyte count than in those with no GI symptoms. The study highlighted that patients with GI symptoms on initial presentation had longer time lag between symptom onset and admission than patients without GI symptoms, reflecting the likely diagnostic delay as typical respiratory symptoms were not predominant on presentation. A study by Zhang et al. investigated infected patients and found the presence of SARS-CoV-2 in anal swabs and blood [9]. The number of positive anal swabs was higher than the number of positive oral swabs in the later stages of the disease, suggesting viral shedding and thereby facilitating fecal-oral transmission of the virus.

The possible mechanism of liver tissue injury in COVID-19 may occur via the up-regulation of angiotensin-converting enzyme 2 (ACE2) expression in liver tissue [10-11]. This increased ACE2 expression is secondary to bile duct epithelial cell-derived hepatocyte proliferation [10-11]. This finding is further supported by an immunofluorescence study conducted by Xiao et al. that elucidates how the ACE-2 protein cell receptor for SARS-CoV-2 is also expressed in gastric, duodenal and rectal epithelial cells [12]. The predominant GI symptoms in our patient may, therefore, have been secondary to direct GI tract invasion in addition to an indirect inflammatory mechanism.

Lastly, gut microbiota plays a key role in the physiology of immune response [9, 13-14]. The virus could disturb the gut flora, which would explain the pathophysiology of GI symptoms in SARS-CoV-2 infected patients [9]. The term “gut-lung axis” has been recently used and studies show that changes in gut flora can affect the respiratory tract and vice versa through a common mucosal immune regulation pathway [9, 13-14].

## Conclusions

Overall, it is imperative for clinicians to screen patients with GI symptoms for COVID-19 in an effort to manage and contain the SARS-CoV-2 virus efficiently. Currently, medical practitioners are screening patients for COVID-19 based primarily on the presence of fever and respiratory symptoms. Our patient is among the few accounts within the United States for which GI manifestations were her only presenting symptom. It is paramount that additional symptomatology with respect to the GI tract is noted as well given the known predilection for viral shedding and fecal-oral transmission. During this global health emergency, the identification of at-risk individuals through an improved understanding of viral presentation can enable immediate recognition and treatment before further transmission occurs.
